# Characterizing the binding interaction of astilbin with bovine serum albumin: a spectroscopic study in combination with molecular docking technology

**DOI:** 10.1039/c7ra13272g

**Published:** 2018-02-13

**Authors:** Jianli Liu, Yonglin He, Dan Liu, Yin He, Zhipeng Tang, Hong Lou, Yapeng Huo, Xiangyu Cao

**Affiliations:** School of Life Science, Liaoning University 66 Chongshan Middle Road Shenyang Liaoning P. R. China caoxiangyu@lnu.edu.cn +86 024 62202913

## Abstract

Astilbin (ASN) is a flavonoid compound isolated from the rhizome of *Smilax china* L. (Smilacaceae). It has many bioactivities, such as selective immunosuppression, antioxidant, anti-hepatic injury, *etc.*, and is widely used in traditional Chinese medical treatments. The interaction of ASN with bovine serum albumin (BSA) was studied in a physiological buffer (pH = 7.40) using multi-spectroscopic techniques in combination with molecular docking methods. UV-vis absorption measurements proved that a ASN–BSA complex could be formed. Fluorescence data revealed that ASN could strongly quench the intrinsic fluorescence of BSA in terms of a static quenching procedure. The process of binding was spontaneous and the binding occurred mainly through hydrogen bonding and van der Waals forces. The distance *r* between donor (BSA) and acceptor (ASN) was calculated to be 4.80 nm based on Förster's non-radiative energy transfer theory. The binding constant (*K*_a_ = 7.31 × 10^4^ mol L^−1^) and the number of binding sites (*n* ≈ 1) at 298 K suggested that ASN only occupied one site in BSA with high affinity. Moreover, the results of molecular docking indicated that ASN was more likely to be located in site I (sub-domain IIA) of BSA. The results of synchronous fluorescence and three-dimensional fluorescence spectra showed that ASN induced conformational changes of BSA. The findings would be beneficial for research on the transportation, distribution and some important bioactivities of ASN in the human body.

## Abstract 2

The interaction of astilbin with bovine serum albumin was confirmed by multi-spectroscopic techniques and molecular docking methods.

## Graphical abstract

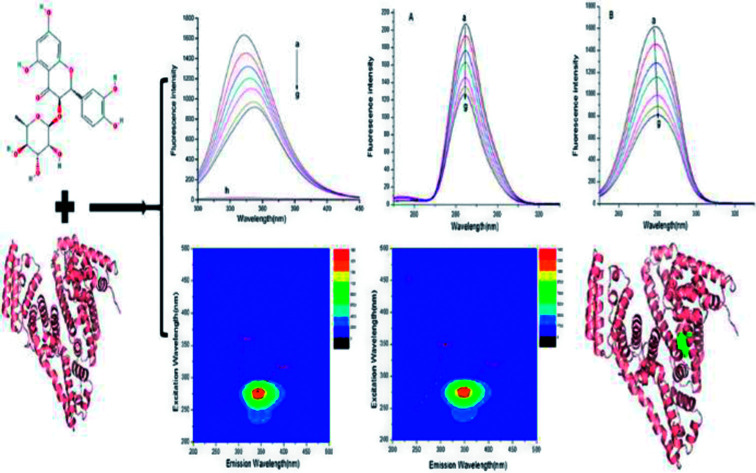

## Introduction

**Fig. 1:**
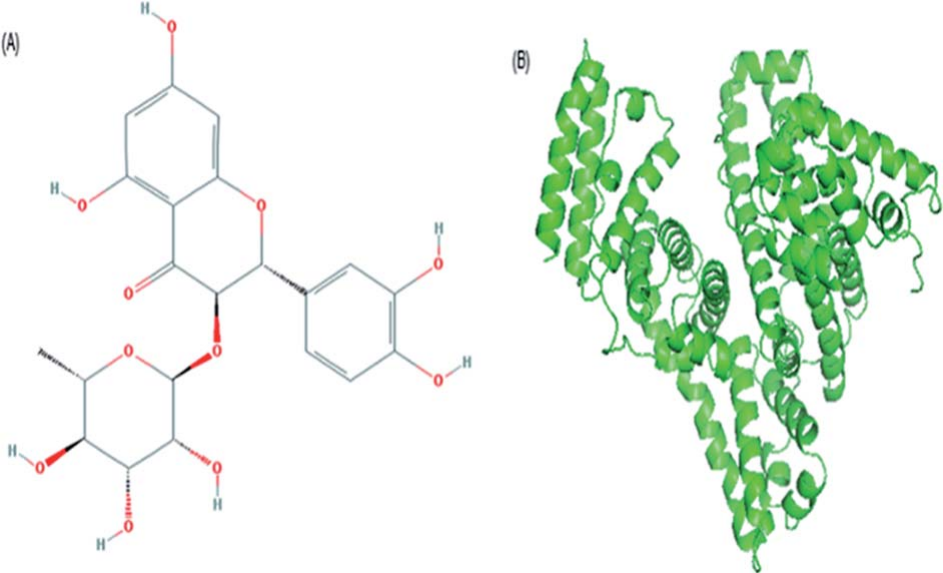
The structure of ASN (A) and BSA (B).

Astilbin (ASN) is a small molecule flavonoid compound isolated from the rhizome of *Smilax china* L. (Smilacaceae), as shown in Fig. 1(A).^1^ It possesses many biological activities, such as anti-inflammatory, antimicrobial, anti-arthritic, antioxidant, anti-hepatic injury, anti-renal injury, antiedematogenic and antinociceptive effects, and is widely used in traditional Chinese medical treatments.^2–5^

Serum albumin (SA) is the most abundant protein in blood plasma with a high affinity for a wide variety of metabolites and drugs, and plays a key role in the maintenance of the blood pH and plasma colloid osmotic pressure.^6^ Moreover, the binding of small drug molecules with SA can affect their absorption, transport and distribution *in vivo*.^7^ It is generally accepted that the strong binding of drug with SA can decrease the concentration of free drug in plasma, while weak binding can lead to a short lifetime or poor distribution of the drug. Therefore, it is essential to study the binding interaction of drug molecules with SA, which has been an interesting research field in clinical medicine, chemistry, life sciences and chemicobiology.^8^

Bovine serum albumin (BSA, Fig. 1(B)) is monomer protein that has 583 amino acid residues, containing three homologous helical domains, known as I (1–195), II (196–383) and III (384–583). Each domain is divided into two sub-domains (A and B). There are two major drug binding sites in the sub-domains IIA (site I) and IIIA (site II). BSA has two tryptophan residues, which are named as Trp134 and Trp213. Trp134 is located on the surface of the molecule, and Trp213 lies within a hydrophobic binding pocket of the protein. It is widely used as a model protein to study the binding interaction of drug with protein because of about 76% structural similarity with human SA and is experimentally well accessible.^9–12^

The main purpose of this work was to systematically investigate the interaction of ASN with BSA under simulated physiological conditions. The quenching mechanisms, binding constants, number of binding sites, thermodynamic parameters were characterized by fluorescence approach. The conformational and micro-environment changes of BSA in the presence of ASN were estimated by synchronous fluorescence and three-dimensional fluorescence spectra. The binding active sites between ASN and BSA was studied by molecular docking. In addition, the binding forces and energy transfer were also discussed. The findings would be beneficial for the research on the transportation, distribution and some important bioactivities of ASN in human body.

## Experimental section

### Chemicals and reagents

BSA (purity > 96%) was purchased from Gen-View Scientific INC. (USA) and used without further purification. Astilbin (purity ≥ 98%) was purchased from Chengdu Must Bio-Technology CO., LTD (Chengdu, China). BSA stock solution was prepared at a concentration of 6 × 10^−5^ mol L^−1^ with 0.01 mol L^−1^ phosphate-buffered saline (PBS; pH = 7.40). ASN solution was prepared at a concentration of 1 × 10^−2^ mol L^−1^ with DMSO solution and diluted with PBS to obtain 6 × 10^−4^ mol L^−1^ stock solution. All reagents were analytical grade and all stock solutions were kept in the dark at 4 °C.

### Fluorescence measurements

All fluorescence measurements were conducted utilizing a Hitachi F-7000 type Fluorescence Spectrophotometer (Hitachi High-Technologies Co., Ltd., Tokyo, Japan). The fluorescence spectra at two temperatures (298 and 310 K) were recorded from 200 to 500 nm with an excitation wavelength of 280 nm. The excitation and emission slit widths were 5 nm.^13,14^ In the measurements, BSA was diluted with 0.01 mol L^−1^ PBS to obtain the final concentration of 6 × 10^−6^ mol L^−1^. Each time, a certain amount of 6 × 10^−4^ mol L^−1^ ASN was added into 6 × 10^−6^ mol L^−1^ BSA. The concentrations of ASN were changed from 0 to 2.4 × 10^−5^ mol L^−1^ with a step of 0.4 × 10^−5^ mol L^−1^ during the interaction.

### Synchronous fluorescence measurements

Synchronous fluorescence spectra of BSA with different ASN concentrations at 298 K were received and the wavelength difference (Δ*λ*) between excitation wavelength (*λ*_ex_) and emission wavelength (*λ*_em_) was respectively fixed at 15 and 60 nm over the wavelength range of 200–500 nm.^15^

### Three-dimensional fluorescence measurements

The three-dimensional (3D) fluorescence spectra at 298 K were recorded at excitation wavelength ranging from 200 to 500 nm and emission wavelength ranging from 200 to 500 nm. The scan speed was set at 1200 nm min^−1^.^16^ The final concentration of BSA was fixed at 6 × 10^−6^ mol L^−1^, while the final concentration of ASN was 1.2 × 10^−5^ mol L^−1^.

### UV-vis absorption experiments

The UV-vis absorption spectra at 298 K were recorded on a UV-2700 spectrophotometer (Shimadzu Co., Kyoto, Japan) over a wavelength range of 190–500 nm.^17^ The final concentration of BSA was fixed at 0.6 × 10^−5^ mol L^−1^. The concentrations of ASN were varied from 0 to 3 × 10^−5^ mol L^−1^ with a step of 0.6 × 10^−5^ mol L^−1^.

### Molecular docking

The crystal structure of BSA (PDB ID:4F5S) was taken from protein data bank (http://www.rcsb.org/pdb/home/home.do). The geometry of ASN (CID:119258) was obtained from National Center for Biotechnology Information (http://pubchem.ncbi.nlm.nih.gov/). Molecular docking simulations were performed using Autodock 4.2 program through Autodock tools 1.5.6 to identify potential binding sites and binding energy.^8^ The possible conformations of ASN–BSA complex were calculated by Lamarckian genetic algorithm. The grid map was set to 80, 80, and 80 along *X*-, *Y*-, and *Z*-axis with a grid-point spacing of 0.4 Å. The running times of genetic algorithm was set at 100, and the maximum number of evaluations and the maximum number of generations were set to 2 500 000 and 27 000. All other parameters were set at the default value. The conformer with the lowest binding energy was further analysed using PyMOL Molecular Graphics System.^9^

## Results and discussion

### Fluorescence quenching studies

**Fig. 2:**
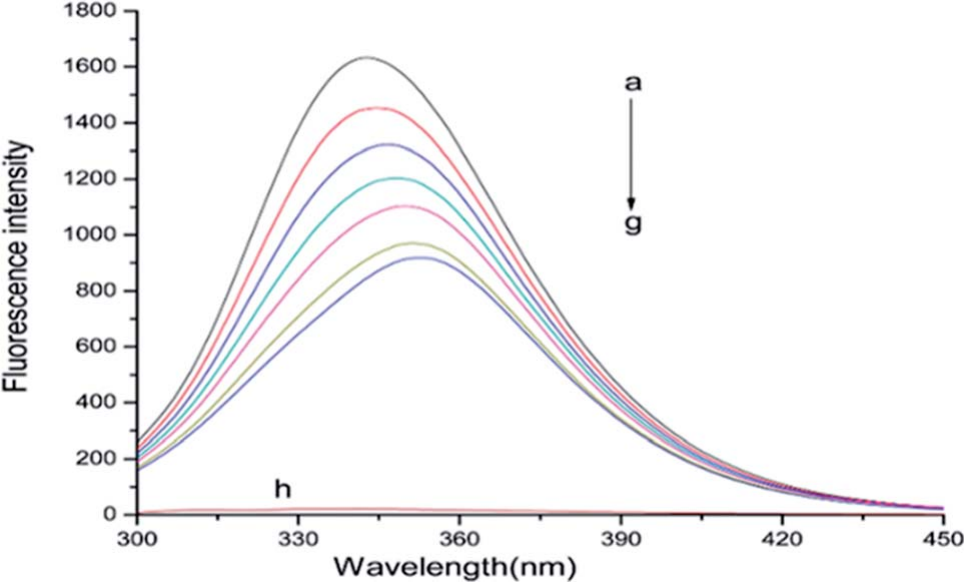
Fluorescence spectra of ASN–BSA solutions with ASN concentrations (a–g) from 0 to 2.4 × 10^−5^ mol L^−1^ at 0.4 × 10^−5^ mol L^−1^ intervals. Curve h showed the emission spectrum of ASN only at the concentration of 0.6 × 10^−5^ mol L^−1^, *C*_BSA_ = 0.6 × 10^−5^ mol L^−1^, *T* = 298 K.

The binding mechanisms, binding constants, binding sites between BSA and drugs can be measured by fluorescence spectroscopy technique.^18^ BSA has endogenous fluorescence because it contains tryptophan, tyrosine and phenylalanine residues. Commonly, the intrinsic fluorescence of BSA is almost exclusively contributed by tryptophan (Trp) and is the most sensitive residue to the changes in the micro-environment, which is widely used as a endogenous fluorescent probe to research the binding interaction between proteins and drugs.^19^ The fluorescence emission spectra of BSA with various concentrations of ASN at 298 K were shown in Fig. 2. It could be seen that BSA exhibited a strong fluorescence emission peak at 344 nm when excited at 280 nm. The fluorescence intensity of BSA gradually decreased with the increasing concentrations of ASN, indicating that the binding of ASN to BSA quenched the intrinsic fluorescence of BSA.^20^ In addition, a visible red shift (7 nm) at the maximum *λ*_em_ was observed in the binding process. These changes indicated that Trp residues may have transferred to a more hydrophilic environment. Bin Tang *et al.* investigated the binding reactions between human serum albumin (HSA) and chlorogenic acid (CA) and its two positional isomers, neochlorogenic acid (NCA) and cryptochlorogenic acid (CCA), also getting the similar results.^21^

Generally, the fluorescence quenching mechanisms can be classified as static quenching, dynamic quenching or non-radiative energy transfer.^22^ Dynamic quenching is collisional quenching, which can be enhanced by increasing temperature while static quenching can form a new ground-state complex between the fluorophores and quenchers.^23^

The analysis of the quenching mechanisms were conducted by using the Stern–Volmer equation to the fluorescence quenching data.^24^

1 *F*_0_/*F* = 1 + *K*_sv_[Q] = 1 + *k*_q_*τ*_0_[Q]

where *F* and *F*_0_ respectively denote the fluorescence intensity of BSA with and without quencher (ASN), *K*_sv_ is the Stern–Volmer dynamic quenching constant, [Q] is the concentration of the quencher, *k*_q_ is the quenching rate constant, and *τ*_0_ is the average fluorescence lifetime of biomolecule in the absence of quencher (*τ*_0_ = 10^−8^ s).^25^

**Fig. 3:**
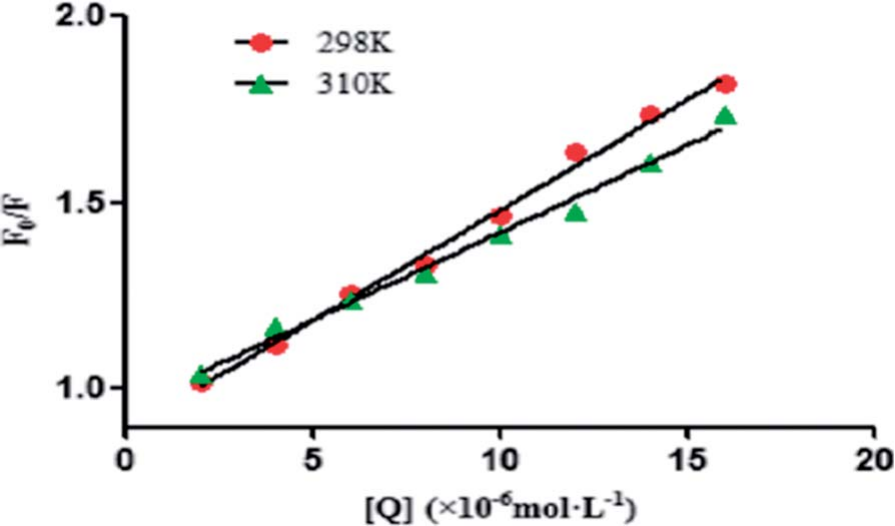
Stern–Volmer plots for the quenching of BSA by ASN at 298 and 310 K.

The plots of *F*_0_/*F* against [Q] of ASN quencher with BSA at different temperatures (298 and 310 K) were shown in Fig. 3, indicating the good linearity relationship. The values of *K*_sv_ could be calculated from the slope of curves. It could be seen that the *K*_sv_ values decreased with the increasing temperature and the values of *k*_q_ were greater than the maximum scattering collision quenching rate constant (2.0 × 10^10^ L mol^−1^ s^−1^) from Table 1 and Fig. 3, which indicated that the fluorescence quenching mechanism was the static quenching.^26^

**Table 1:** Stern–Volmer equation constants for the interaction of ASN with BSA at 298 and 310 K

*T* (K)	*K*_sv_ (×10^4^ mol L^−1^)	*k*_q_ (×10^12^ L mol^−1^ s^−1^)	*R* a
298	3.39	3.39	0.9913
310	2.64	2.64	0.9902

a *R* is the correlation coefficient for the *K*_sv_ values.

For the static quenching interaction, the number of binding sites (*n*) and the binding constants (*K*_a_) can be measured by the following equation:^27^

**Fig. 4:**
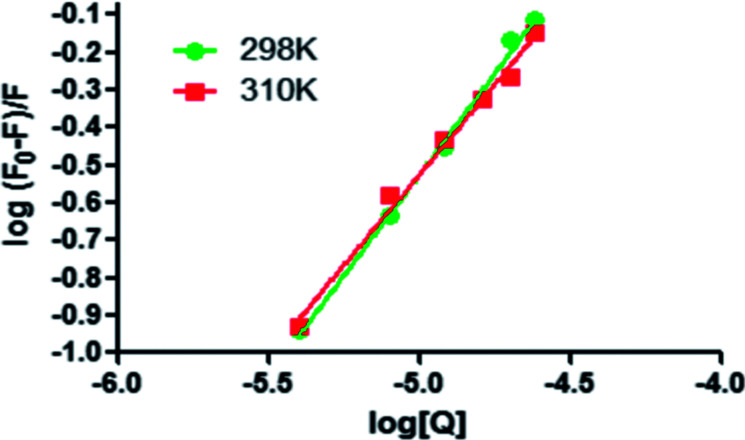
Modified Stern–Volmer plots for the binding of ASN with BSA at 298 and 310 K.

2 log[(*F*_0_ − *F*)/*F*] = log *K*_a_ + *n* log[Q]

where *F*_0_, *F* and [Q] are the same as in eqn (1), *K*_a_ is the binding constant and *n* is the number of binding sites. By using the intercept and slope of the above equation log[(*F*_0_ − *F*)/*F*] *versus* log[Q] as shown in Fig. 4, the values of *K*_a_ and *n* can be measured. The results were shown in Table 2. The calculated *K*_a_ and *n* values decreased with the increasing temperature, which was further proved that the quenching mechanism was static quenching.^28^ Moreover, *n* values were calculated to approximately equal 1, indicating that there was one binding site on the BSA for ASN binding. The *K*_a_ values were in the magnitude of 10^4^ mol L^−1^, indicating the binding between ASN and BSA with high affinity.

**Table 2:** The binding constants *K*_a_ and the relative thermodynamic parameters for the interaction between ASN and BSA at 298 and 310 K

*T* (K)	*K*_a_ (×10^4^ mol L^−1^)	*n*	*R* a	Δ*H* (kJ mol^−1^)	Δ*G* (kJ mol^−1^)	Δ*S* (J mol^−1^ K^−1^)
298	7.31	1.08	0.9965	−111.99	−27.75	−282.68
310	1.27	0.92	0.9914		−24.36	

a *R* is the correlation coefficient for the *K*_a_ values.

The binding interaction between small drug molecules and biological macromolecules can be mainly driven by one or more of the several binding forces which are called as hydrogen bonding, hydrophobic interaction, van der Waals force and electrostatic interaction.^29^ In order to explain the binding interaction between ASN and BSA, the thermodynamic parameters were obtained from eqn (3)–(5).^10^ If the temperature only slightly changes, the enthalpy change (Δ*H*) can be regarded as a constant. Making use of the binding constants at two temperatures, the free energy change (Δ*G*) can be calculated by the following equation:

3 Δ*G* = −*RT* ln *K*

where *K* is the binding constant, *T* is the experimental temperature and *R* is the gas constant.

The entropy change (Δ*S*) and enthalpy change (Δ*H*) can be calculated from the eqn (4) and (5):

4 ln(*K*_2_/*K*_1_) = (1/*T*_1_ − 1/*T*_2_) × Δ*H*/*R*

where *K*_1_ and *K*_2_ are the binding constant at two temperatures *T*_1_ (298 K) and *T*_2_ (310 K), respectively.

5 Δ*S* = (Δ*H* − Δ*G*)/*T*

The results of thermodynamic parameters were shown in Table 2. The negative value of free energy change (Δ*G*) indicated that the interaction between ANS and BSA was a spontaneous process. The values of enthalpy change (Δ*H*) and entropy change (Δ*S*) were negative, proving that the interaction between ASN and BSA were mainly driven by hydrogen bonding and van der Waals force.^30^

### Energy transfer between BSA and ASN

Förster non-radiative energy transfer theory is used for quantifying the distance and energy transfer efficiency between the donor and acceptor when the fluorescence spectrum of donor and the UV-vis absorption spectrum of acceptor overlap each other.^31^ It can be calculated by the following equations:^30^

**Fig. 5:**
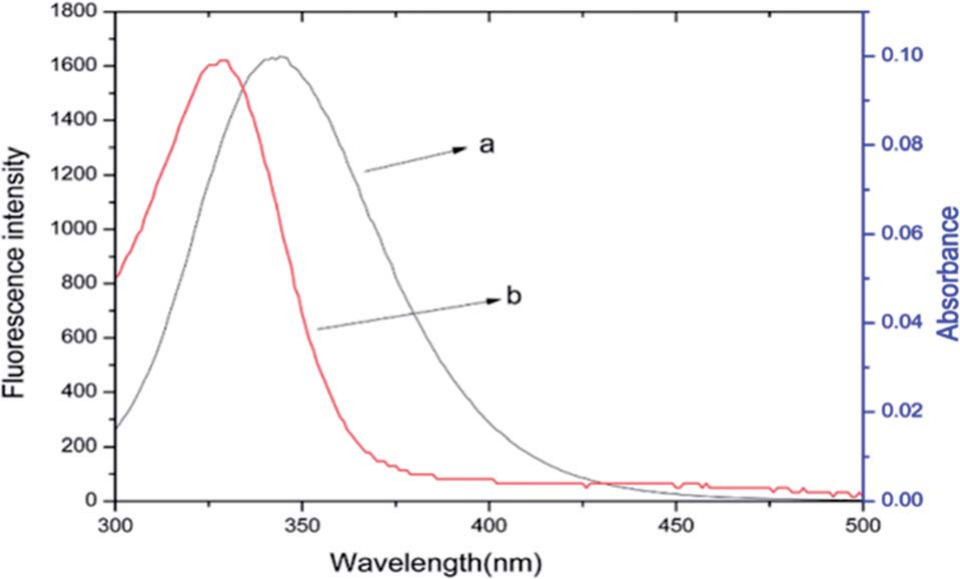
The spectral overlaps of the fluorescence spectrum of BSA (a) with the UV-visible absorption spectrum of ASN (b), *C*_BSA_ = *C*_ASN_ = 6 × 10^−6^ mol L^−1^, *T* = 298 K.

6 *E* = 1 − *F*/*F*_0_ = *R*_0_^6^/(*R*_0_^6^ + *r*^6^)

where *r* is the distance between acceptor and donor, *R*_0_ is the critical distance for transfer efficiency of 50%, which can be calculated by eqn (7):

7 *R*_0_^6^ = 8.8 × 10^−25^*k*^2^*n*^−4^*ΦJ*

where *k*^2^ is the spatial orientation factor of the dipole, *k*^2^ = 2/3. *n* is the refractive index of medium, *n* = 1.336. *Φ* is the fluorescence quantum yield of the donor, *Φ* = 0.118 for BSA. *J* is the overlap integral of the emission spectrum of the donor and absorption spectrum of the acceptor, which can be calculated by the following equation:

8 *J* = ∑*F*(*λ*)*ε*(*λ*)*λ*^4^Δ*λ*/∑*F*(*λ*)Δλ

where *F*(*λ*) and *ε*(*λ*) are the fluorescence intensity of the donor and the molar extinction coefficient of the acceptor at wavelength *λ*, respectively. The *J* value can be calculated by integrating overlapped portion of fluorescence emission spectrum of BSA with UV-vis absorption spectrum of ASN as shown in Fig. 5, which was 9.54 × 10^−14^ cm^3^ L^−1^ mol^−1^. The values of *E*, *R*_0_ and *r* were 0.14, 3.57 nm and 4.80 nm, respectively. The binding distance *r* < 7 nm and 0.5*R*_0_ < *r* < 1.5*R*_0_, which indicated that energy transfer from BSA to ANS occurred with high probability. Zhang *et al.* investigated the binding interaction of maltol with BSA, also getting the similar results.^32^

### Synchronous fluorescence spectra

**Fig. 6:**
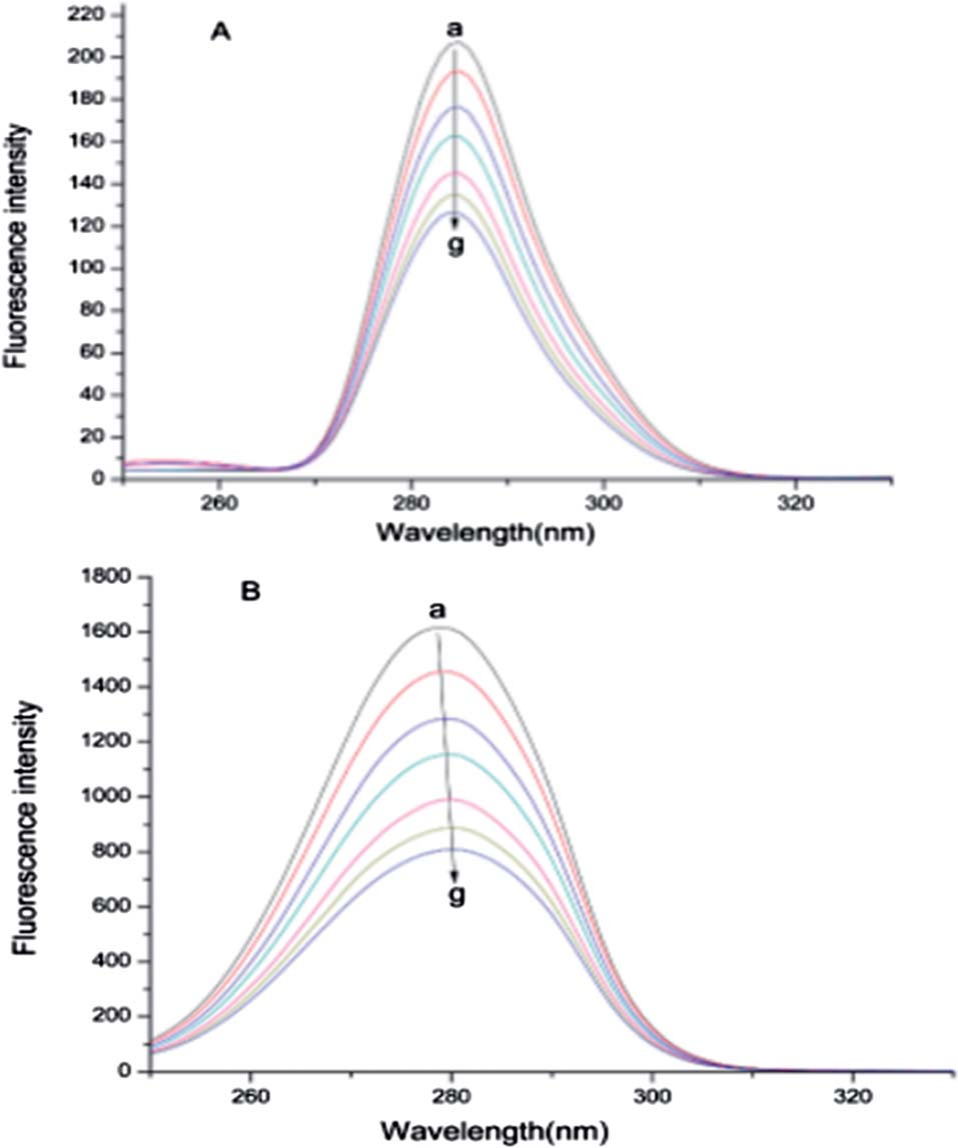
Synchronous fluorescence spectra of BSA in the absence and presence of different concentrations of ASN. From (a–g), the concentrations of ASN were varied from 0 to 2.4 × 10^−5^ mol L^−1^ at 0.4 × 10^−5^ mol L^−1^ intervals, (A) Δ*λ* = 15 nm, (B) Δ*λ* = 60 nm, *C*_BSA_ = 0.6 × 10^−5^ mol L^−1^, *T* = 298 K.

The synchronous fluorescence spectrum can respectively offer characteristic information about Tyr residues or Trp residues of BSA. When the intervals (Δ*λ*) value between the excitation and emission wavelength are set at 15 or 60 nm,^33^ which means that the changes of micro-environment around Tyr and Trp residues can be measured by observing the maximum emission wavelength shift of synchronous fluorescence spectra. In general, the conformation of BSA is closely related to the micro-environment surrounding Tyr and Trp residues.^12^ As shown in Fig. 6, the fluorescence intensity of Tyr and Trp residues gradually decreased with the addition of ASN. A slight red shift (2 nm) was found at the maximum *λ*_em_ of Trp residues and the maximum *λ*_em_ of Tyr residues almost did not change, indicating that the slight decrease in the hydrophobicity surrounding Trp residues but almost no change in the hydrophobicity surrounding Tyr residues. These results revealed that the conformation of BSA was changed. Jiang *et al.* studied the binding interaction of ramipril with BSA and also got the similar results.^12^

### Three-dimensional fluorescence spectra

**Fig. 7:**
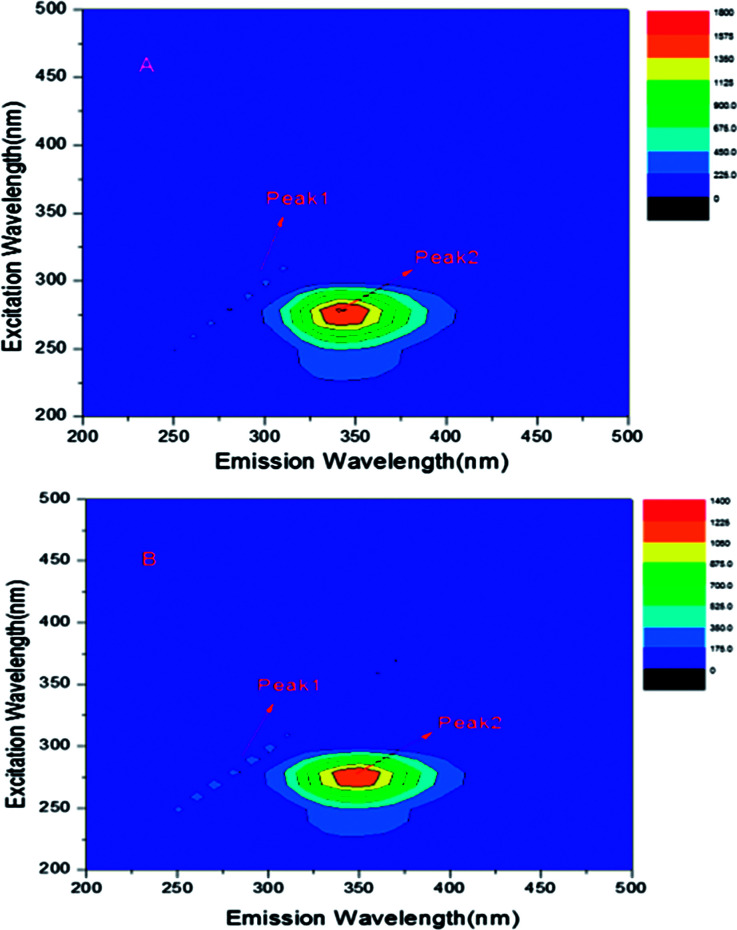
(A) Three-dimensional fluorescence spectra of free BSA and the system of ASN–BSA (B), *C*_BSA_ = 0.6 × 10^−5^ mol L^−1^, *C*_ASN_ = 1.2 × 10^−5^ mol L^−1^, *T* = 298 K.

Three-dimensional (3-D) fluorescence spectroscopy can be used to observe the conformational changes of proteins when the binding interaction between drugs and proteins occur.^13^ Therefore, the three-dimension fluorescence spectra of free BSA and ASN–BSA complex was measured as shown in Fig. 7, and the corresponding parameters were listed in Table 3. Peak 1 was the Rayleigh scattering peak (*λ*_ex_ = *λ*_em_). Peak 2 indicated that the spectral characteristics of Tyr and Trp residues on BSA and the fluorescence intensity and the maximum emission wavelength were closely related to the polarity of the micro-environment surrounding Tyr and Trp residues.^15^ The results indicated that the fluorescence intensity of peak 2 decreased with the addition of ASN. The maximum emission wavelength showed the red shift (from 345 to 350 nm), revealing that the polarity of the micro-environment around Tyr and Trp residues and the conformation of BSA were changed. Dong *et al.* studied the binding interaction of antimalarial artemether (AMT) with BSA, also obtaining the similar results.^19^

**Table 3:** The 3-D fluorescence spectral characteristic parameters of free BSA and the ASN–BSA complex, *C*_BSA_ = 0.6 × 10^−5^ mol L^−1^, *C*_ASN_ = 1.2 × 10^−5^ mol L^−1^, *T* = 298 K

System	Peak 2 position *λ*_ex_/*λ*_em_ (nm nm^−1^)	Fluorescence intensity
Free BSA	280/345	1614
ASN–BSA	280/350	1221

### UV-vis absorption spectra

**Fig. 8:**
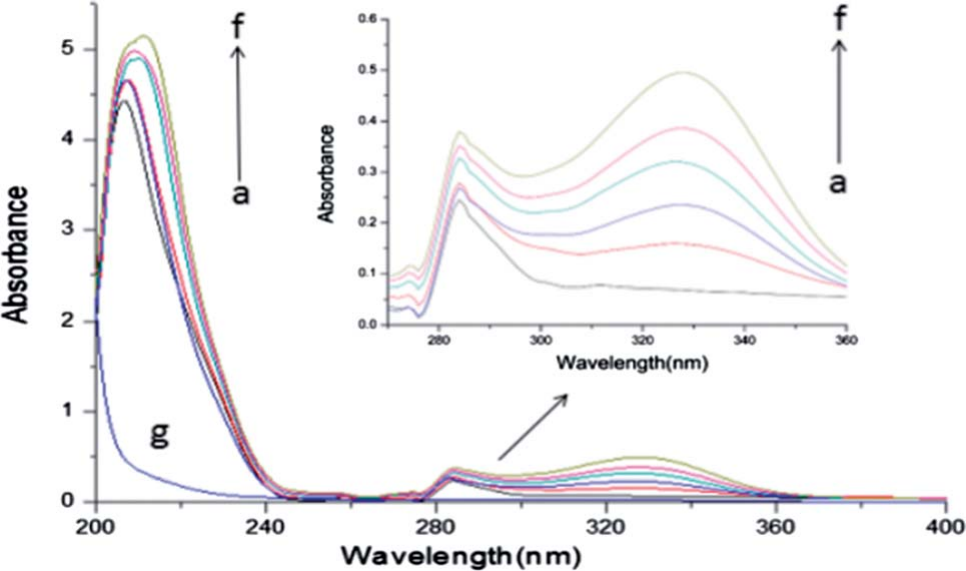
The UV-vis absorption spectra of BSA in the absence and presence of different concentrations of ASN. From (a–f), the concentrations of ASN were varied from 0 to 3.0 × 10^−5^ mol L^−1^ at 0.6 × 10^−5^ mol L^−1^ intervals. Curve g showed the UV-vis absorption spectra of ASN only at the concentration of 0.6 × 10^−5^ mol L^−1^, *C*_BSA_ = 0.6 × 10^−5^ mol L^−1^, *T* = 298 K.

UV-vis absorption measurement is a very effective method to predict the complex formation and the conformational changes of proteins during the binding interaction between small drug molecules and proteins.^26^ The UV-vis absorption spectra of BSA with different ASN concentrations were shown in Fig. 8, indicating that there were mainly two absorption bands. A weak band around 280 nm belongs to the π → π* transition of the aromatic amino acids such as Trp, Tyr, and Phe, and a strong band around 210 nm reflects the framework conformation of BSA. In addition, the absorption peak at 328 nm was the free ASN, and its intensity increased with the increasing ASN concentrations. The absorption peak intensity at near 210 and 280 nm increased with increasing the concentrations of ASN. Moreover, the absorption peak arose the visible red shift at near 210 nm. These results proved that complex formation between ASN and BSA and led to the conformational changes of BSA. Zhou *et al.* conducted the investigation the binding interaction of clonazepam with BSA, which got the similar results.^7^

### Molecular docking

**Fig. 9:**
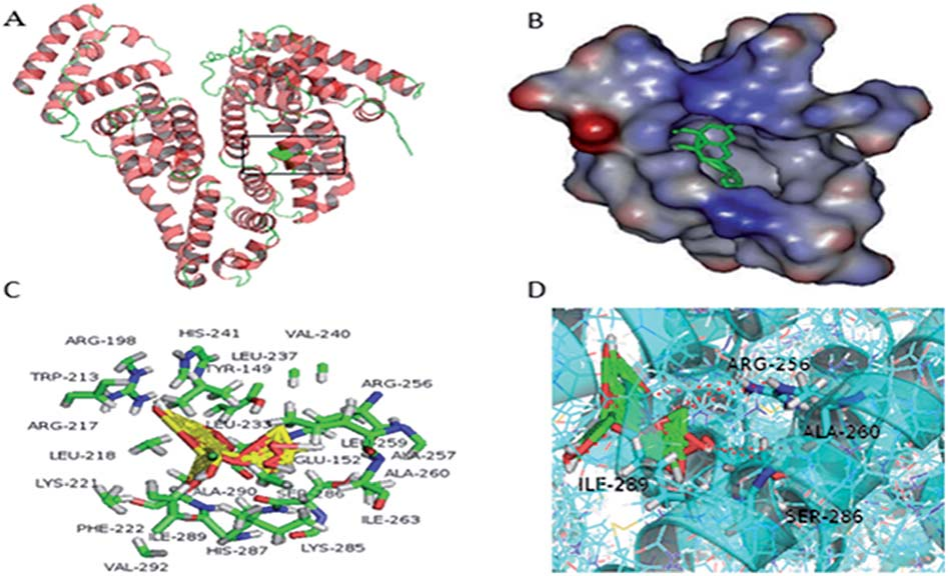
(A) The minimum energy conformation of ASN–BSA complex obtained from molecular docking. BSA was represented by a ribbon structure and ASN was represented by stick model. (B) ASN inserted into the hydrophobic cavity in site I of BSA. (C) ASN was surrounded by amino acid residues of BSA in 5 Å. (D) Hydrogen bonding interaction of ASN with some amino acid residues of BSA.

The binding interaction forces and preferred binding sites for ASN binding to BSA can be measured by molecular docking methods.^34^ It was evident from Fig. 9, ASN was inserted into the hydrophobic pocket in sub-domain IIA (site I) of BSA and was surrounded by many hydrophobic residues, including Trp-213, Leu-218, Leu-233, Leu-237, Leu-259, Phe-222, Val-240, Val-292, Ile-263, Ile-289, Ala-257, Ala-260 and Ala-290, which also suggested that there was hydrophobic interaction between ASN and BSA. Moreover, there were six hydrogen bondings with Arg-256, Ala-260, Ser-286 and Ile-289. Based on these molecular docking information in combination with thermodynamic parameters, it could be implied that hydrogen bonding and van der Waals force played a dominate role for ASN binding to BSA. Shen *et al.* studied the binding interaction of clonazepam with BSA, also obtaining the similar results.^7^

## Conclusions

In this study, the interaction of ASN with BSA were carried out by multi-spectroscopic techniques in combination with molecular docking methods. The experimental results revealed that ASN could quench the endogenous fluorescence of BSA. The quenching mechanism was the static quenching, and the binding process was spontaneous. The binding between ASN and BSA was only a binding site, and was more likely to be located in site I (sub-domain IIA) of BSA. The binding distance was calculated to be 4.80 nm and the binding constants were in the magnitude of 10^4^ mol L^−1^. The main binding interaction forces were hydrogen bonding and van der Waals force and the conformation of BSA was changed by the binding with ASN. This work would provide comprehensive basic information for the binding mode of ASN with BSA, which was useful for researching pharmacokinetics such as drug metabolism, transportation, distribution and excretion. In addition, it was very beneficial to research the mechanism of many bioactivities about ASN in human body. However, different research methods should be used to modify the ASN structure so as to make it better be transported and distributed in human body and played a good biological activity.

## Conflicts of interest

There are no conflicts to declare.

## Supplementary Material

## References

[cit1] Kong G. Q., Huang X., Wang L. P., Li Y., Sun T., Han S. S., Zhu W. W., Ma M. M., Xu H. X., Li J. K., Zhang X. H., Liu X. Y., Wang X. Z. (2016). Int. Immunopharmacol..

[cit2] Diao H. L., Kang Z. C., Han F., Jiang W. L. (2014). Food Chem. Toxicol..

[cit3] Wang M., Zhao J., Zhang N., Chen J. H. (2016). Biomed. Pharmacother..

[cit4] Di T. T., Ruan Z. T., Zhao J. X., Wang Y., Liu X., Wang Y., Li P. (2016). Int. Immunopharmacol..

[cit5] Ding Y. B., Liang Y., Deng B., Qiao A. H., Wu K. Y., Xiao W. M., Gong W. J. (2014). Biochem. Biophys. Res. Commun..

[cit6] Shi J. H., Zhou K. L., Lou Y. Y., Pan D. Q. (2018). Spectrochim. Acta, Part A.

[cit7] Lou Y. Y., Zhou K. L., Pan D. Q., Shen J. L., Shi J. H. (2017). J. Photochem. Photobiol., B.

[cit8] Shen G. F., Liu T. T., Wang Q., Jiang M., Shi J. H. (2015). J. Photochem. Photobiol., B.

[cit9] Wang Q., Huang C. R., Jiang M., Zhu Y. Y., Wang J., Chen J., Shi J. H. (2016). Spectrochim. Acta, Part A.

[cit10] Esfahlan A. J., Azar V. P. (2016). Food Chem..

[cit11] Uppuluri K. B., Ayaz Ahmed K. B., Jothi A., Veerappan A. (2016). J. Mol. Liq..

[cit12] Shi J. H., Pan D. Q., Jiang M., Liu T. T., Wang Q. (2016). J. Photochem. Photobiol., B.

[cit13] Nair M. S. (2015). J. Photochem. Photobiol., B.

[cit14] Zhang G. W., Ma Y. D. (2013). Food Chem..

[cit15] Shen H. B., Gu Z. Q., Jian K., Qi J. (2013). J. Pharm. Biomed. Anal..

[cit16] Abdelhameed A. S., Alanazi A. M., Bakheit A. H., Darwish H. W., Ghabbour H. A., Darwish I. A. (2017). Spectrochim. Acta, Part A.

[cit17] Sun Q. M., Yang H. Q., Tang P. X., Liu J. Y., Wang W., Li H. (2018). Food Chem..

[cit18] Sajid Ali M., Al-Lohedan H. A. (2017). J. Mol. Liq..

[cit19] Shi J. H., Pan D. Q., Wang X. X., Liu T. T., Jiang M., Wang Q. (2016). J. Photochem. Photobiol., B.

[cit20] Xu L., Hu Y. X., Li J., Liu Y. F., Zhang L., Ai H. X., Liu H. S. (2017). J. Photochem. Photobiol., B.

[cit21] Tang B., Huang Y. M., Ma X. L., Liao X. X., Wang Q., Xiong X. N., Li H. (2016). Food Chem..

[cit22] Ao J. J., Gao L., Yuan T., Jiang G. F. (2015). Chemosphere.

[cit23] Kumari M., Maurya J. K., Singh U. K., Khan A. B., Ali M., Singh P., Patel R. (2014). Spectrochim. Acta, Part A.

[cit24] Yasmeen S., Riyazuddeen (2017). Thermochim. Acta.

[cit25] Fu L., Sun Y. Q., Ding L. N., Wang Y. Y., Gao Z., Wu Z., Wang S. M., Li W., Bi Y. F. (2016). Food Chem..

[cit26] Lou Y. Y., Zhou K. L., Shi J. H., Pan D. Q. (2017). J. Photochem. Photobiol., B.

[cit27] Teng Y., Liu R. T., Li C., Xia Q., Zhang P. J. (2011). J. Hazard. Mater..

[cit28] Raza M., Ahmad A., Yue F., Khan Z., Jiang Y., Wei Y., Raza S., He W. W., Khan F. U., Peng Y. Q. (2017). J. Photochem. Photobiol., B.

[cit29] Sarkar M., Paul S. S., Mukherjea K. K. (2013). J. Lumin..

[cit30] Shi J. H., Chen J., Wang J., Zhu Y. Y., Wang Q. (2015). Spectrochim. Acta, Part A.

[cit31] Rabbani G., Baig M. H., Jan A. T., Lee E. J., Khan M. V., Zaman M., Farouk A. E., Khan R. H., Choi I. (2017). Int. J. Biol. Macromol..

[cit32] Zhang G. W., Ma Y. D., Wang L., Zhang Y. P., Zhou J. (2012). Food Chem..

[cit33] Zhang G. W., Zhao N., Wang L. (2011). J. Lumin..

[cit34] Wang Y. J., Zhang G. W., Yan J. K., Gong D. M. (2014). Food Chem..

